# ADAR1-mediated RNA-editing of 3′UTRs in breast cancer

**DOI:** 10.1186/s40659-018-0185-4

**Published:** 2018-10-05

**Authors:** Eduardo A. Sagredo, Alejandro Blanco, Alfredo I. Sagredo, Paola Pérez, Gonzalo Sepúlveda-Hermosilla, Fernanda Morales, Bettina Müller, Ricardo Verdugo, Katherine Marcelain, Olivier Harismendy, Ricardo Armisén

**Affiliations:** 1 Center of Excellence in Precision Medicine, Pfizer Chile, Obispo Arturo Espinoza Campos 2526, 7810305 Santiago, Chile; 2 Centro de Investigación y Tratamiento del Cáncer, Facultad de Medicina, Universidad de Chile, Independencia 1027, Santiago, Chile; 3 Servicio de Oncología Médica, Instituto Nacional del Cáncer, Avenida Profesor Zañartu 1010, Santiago, Chile; 4 Programa de Genética Humana, ICBM, Facultad de Medicina, Universidad de Chile, Independencia 1027, Santiago, Chile; 5 Departamento de Oncología Básico Clínica, Facultad de Medicina, Universidad de Chile, Independencia 1027, Santiago, Chile; 6 Moores Cancer Center and Division of Biomedical Informatics, Department of Medicine, School of Medicine, University of California, San Diego, CA USA; 7 Grupo Oncológico Cooperativo Chileno de Investigación GOCCHI, Santiago, Chile

**Keywords:** Breast cancer, ADAR1, 3′UTR, Editing

## Abstract

**Background:**

Whole transcriptome RNA variant analyses have shown that adenosine deaminases acting on RNA (*ADAR*) enzymes modify a large proportion of cellular RNAs, contributing to transcriptome diversity and cancer evolution. Despite the advances in the understanding of *ADAR* function in breast cancer, *ADAR* RNA editing functional consequences are not fully addressed.

**Results:**

We characterized A to G(I) mRNA editing in 81 breast cell lines, showing increased editing at 3′UTR and exonic regions in breast cancer cells compared to immortalized non-malignant cell lines. In addition, tumors from the BRCA TCGA cohort show a 24% increase in editing over normal breast samples when looking at 571 well-characterized UTRs targeted by *ADAR1*. Basal-like subtype breast cancer patients with high level of *ADAR1* mRNA expression shows a worse clinical outcome and increased editing in their 3′UTRs. Interestingly, editing was particularly increased in the 3′UTRs of *ATM*, *GINS4* and *POLH* transcripts in tumors, which correlated with their mRNA expression. We confirmed the role of *ADAR1* in this regulation using a shRNA in a breast cancer cell line (ZR-75-1).

**Conclusions:**

Altogether, these results revealed a significant association between the mRNA editing in genes related to cancer-relevant pathways and clinical outcomes, suggesting an important role of *ADAR1* expression and function in breast cancer.

**Electronic supplementary material:**

The online version of this article (10.1186/s40659-018-0185-4) contains supplementary material, which is available to authorized users.

## Background

The RNA editing process is an essential and widespread mechanism for generating RNA diversity in different organisms. In humans, the main form of RNA editing is the hydrolytic deamination of adenosine, which converts this nucleoside to inosine, catalyzed by the adenosine deaminases acting on RNA (*ADAR*) enzymes [1]. In recent years, several studies have reported alterations of *ADAR1* activity in cancer, where edited sites in coding regions have been associated with cancer progression [2, 3]. Nevertheless, recent studies have revealed key functional consequences of editing function on UTRs, where *ADAR1* activity has been implicated mRNA stability, transcriptome variability and mRNA expression changes, by altering the canonical RNA–RNA and RNA–protein interactions of its targets [4, 5].

Genome-wide studies have revealed that a large proportion of the transcriptome can be modified by these editing enzymes [4, 6–8]. Since inosine is read as guanine by most genetic assays, this editing is referred as A-to-G(I). Regions enriched in A-to-G(I) edited sites are mainly located in intronic and UTRs of the transcript, whereas a limited number of edited sites are located in coding exons leading to non-synonymous changes [5, 9].

In recent years, several studies have reported alterations of *ADAR1* activity in cancer. In multiple cancer models, *ADAR1* overexpression is correlated with oncogenic phenotypes, such as invasion and proliferation, and edited sites in coding regions have been associated with cancer progression [2, 3]. Nevertheless, recent studies have revealed key functional consequences of editing in introns or UTRs, where *ADAR1* activity has been implicated in mRNA splicing [10, 11], RNA localization [12], mRNA stability [5], transcriptome variability [4] and mRNA expression changes [5, 13], by altering the canonical RNA–RNA and RNA–protein interactions of its targets. For instance, A-to-I edited sites in the 3′UTR of cathepsin S mRNA, controls the mRNA stability allowing its interaction with *HuR*, a key mRNA stabilizing RNA binding protein [14]. Moreover, *ADAR1* has been recently shown to interact and stabilize *FAK* mRNA through an edited site located in an intronic region, increasing the mobility and invasion capacities of lung adenocarcinoma cells [15]. This suggests that the A-to-I editing adds an additional level of complexity and plasticity in the function and regulation of the target, with unprecedented implications for diseases, such as cancer.

In this report, we characterized the *ADAR1* editing pattern in 78 breast cancer (BC) and 3 immortalized non-malignant breast cell lines, showing that A to G(I) editing affected transcripts associated to cell cycle and immune response. In addition, we found an increased number of edited sites at 3′UTR and exonic regions in BC cell lines compared to immortalized non-malignant cell lines. Moreover, we found an increase in editing counts at UTRs in tumor samples included in the BRCA TCGA cohort, suggesting that *ADAR1* could modify the expression and/or stability of *ATM*, *GINS4* and *POLH* transcripts. Taken together, this work provides novel insights on the role played by *ADAR1* in the regulation of those edited mRNAs in breast cancer context.

## Results

### ADAR1 expression and activity in breast cell lines

**Fig. 1:**
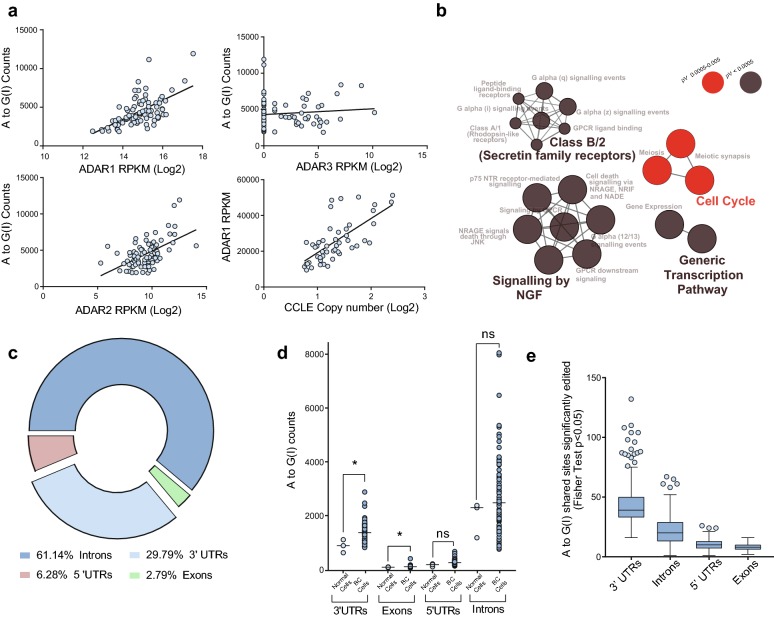
Assessment of *ADAR* activity in BC cell lines. **a** Correlation between A and G(I) variants and *ADAR* isoforms (RPKM) for the 81 breast cells lines included in the PRJNA297219 dataset. Linear regression for normalized data, Spearman Correlation r = 0.585, p < 0.0001 (*ADAR1*); r = 0.476, p < 0.0001 (*ADAR2*), r = 0.07, p < 0.499 (*ADAR3*) and r = 0.697, p < 0.0001(*ADAR1* CCLE CNV). **b** Reactome pathway enrichment associated with A to G(I) variants present in 10% of the BC cell lines. **c** Variant distribution of edited sites for variants present on breast cell lines. **d** Variant counts present in non-malignant MCF10A, MCF12A and 184A1 cell lines and BC cells, according the different transcript regions. **e** Variant counts significantly edited in BC cells distributed according genomic regions, compared to immortalized non-malignant MCF10A, MCF12A and 184A1 cell lines. d*: Kolmogorov–Smirnov p < 0.05, *ns* non-significant differences

We analyzed the transcriptomes from 81 breast cell lines that were publicly available [16], in order to characterize *ADAR1* activity. We found a significant correlation between *ADAR1* expression and the number of A to G(I) sites identified (r = 0.58, p < 0.0001). A to G(I) sites also significantly correlates with *ADAR2* (r = 0.477, p < 0.0001), but not with *ADAR3* expression (r = 0.07, p < 0.499) (Fig. 1a). In addition, we found that *ADAR1* expression significantly correlates with the *ADAR1* gene copy number in each cell line (r = 0.69, p < 0.0001) consistent with previous reports (Fig. 1a) [17, 18].

Previous reports have shown that *ADAR1* targets are enriched for transcripts involved in cell cycle and immune response related processes [4, 5]. To verify these observations in the breast cell lines, we first identified 2872 transcripts containing 9522 high confidence A to G(I) edited sites present in more than 10% (8 cell lines) of the complete dataset (presented in Additional file 1). These edited transcripts are enriched for Cell cycle related pathways (Bonferroni corrected p < 9.9E−7), confirming previous reports [4, 5], among other relevant gene ontologies (Fig. 1b).

RNA editing is not uniformly distributed along the transcripts, showing a large number of edited sites at intronic and 3′UTR regions [4, 19, 20], Similarly, we found a large number of edited sites at those regions (Fig. 1c), where the median number of editing sites for immortalized non-malignant cell lines (n = 3) was 3501 and for BC cells (n = 78) was 4053 A to G(I) sites, not finding significant differences in the total number of edited sites between the immortalized and BC cells (p < 0.2043, Additional file 2: Figure S1A). Interestingly, BC cells presents a significant increase in the number of edited sites located at 3′UTRs (p < 0.0461) and exonic regions (p < 0.0362) (Fig. 1d). In addition, we analyzed the editing levels of all A to G(I) sites shared between BC and immortalized non-malignant cells comparing, at each site, the fraction of reads supporting the edited variant. From the 1131.63 average shared sites between BC and non-malignant cells, 84.52 shared sites presented a significant increase in editing level on BC cells, where 44 sites were located at 3′UTRs, 22.30 sites were in intronic regions, 10.29 at 5′UTRs and 7.92 were in exons (Fig. 1e). Finally, to test if there was a particular region of the transcripts with increased level editing levels on BC cells, we performed a Wilcoxon test for all those shared variants across BC and immortalized non-malignant cell lines. Remarkably, we found that 52/78, 44/78 and 9/78 BC cells present a significant increase in the editing levels for those shared sites located at 3′UTRs compared to 184A1, MCF12 and MCF10A, respectively (Additional file 2: Figure S1B), where 48/234, 28/234 and 4/234 comparisons shown that result on shared sites located at introns, 5′UTRs and exonic regions, respectively (Additional file 3: Table S1), suggesting that in BC cells *ADAR1* present an increased activity on 3′UTRs compared to 184A1 and MCF12 cells.

### Characterization of edited sites from BC tumor transcriptomes in UTRs regions

**Fig. 2:**
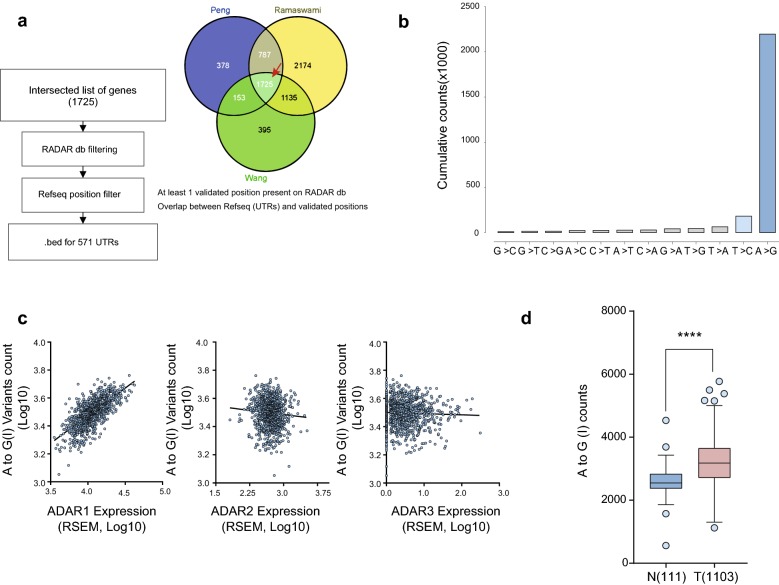
UTRs from the BRCA TCGA cohort show increased editing variants associated to *ADAR1* activity. **a** Workflow for *ADAR1*-target selection, based on the intersection of three independent studies with complementary RNA editing study approaches. **b** Cumulative parameter count across the different possible variants across the UTRs analyzed. **c** Correlation of *ADAR* expression isoforms (RSEM, Log10) and the number of A to G(I) variants for each tumor (Log10). **d** A to G(I) counts in the 571 evaluated UTRs from BRCA TCGA cohort in normal and tumor breast samples. Box and Whiskers plot with Tukey distribution, ****: Kolmogorov–Smirnov p < 0.001. Pearson Correlation analysis for figure (**c**) r = 0.679 p < 0.0001 (*ADAR1*); r = − 0.077 p < 0.01 (*ADAR2*); r = − 0.028 p < 0.3765 (*ADAR3*)

Considering that we observed an increased number of edited sites on 3′UTRs, in the breast cells lines editing analysis, and the growing number of works that have described an *ADAR1* role at UTRs regions [21, 22], we explored the possible implications of RNA editing activity at UTR regions in BC patients. To do that, we called and characterized the RNA variants present in UTRs in 1103 breast tumors and 111 adjacent normal tissues from the BRCA TCGA cohort. The analysis was restricted to 571 UTRs based on the consensus list of genes edited by *ADAR1,* curating the results of three different studies that used alternative approaches to call edited sites in B cell lines [5, 6, 19] (Fig. 2a). Our results shows a clear enrichment of A to G(I) and T to C(I) transitions in BC samples, representing 82.17% and 6.8% of candidate called sites, respectively (Fig. 2b). In order to demonstrate that edited sites detected were related to *ADAR1* overexpression, we correlated the number of edited sites in each tumor with *ADAR1* expression levels. We observed a significant positive correlation between the numbers of A to G(I) edited sites and *ADAR1* expression (r = 0.679, p < 0.0001), a discrete, but significant negative correlation, for *ADAR2* expression (r = − 0.077, p < 0.01), and no correlation for A*DAR3* (r = − 0.028, p < 0.3765) expression levels (Fig. 2c). Furthermore, there was a 1.24 fold change (FC) increase (p < 0.001) in the number of A to G(I) edited sites in tumors compared to normal tissues (Fig. 2d).

**Fig. 3:**
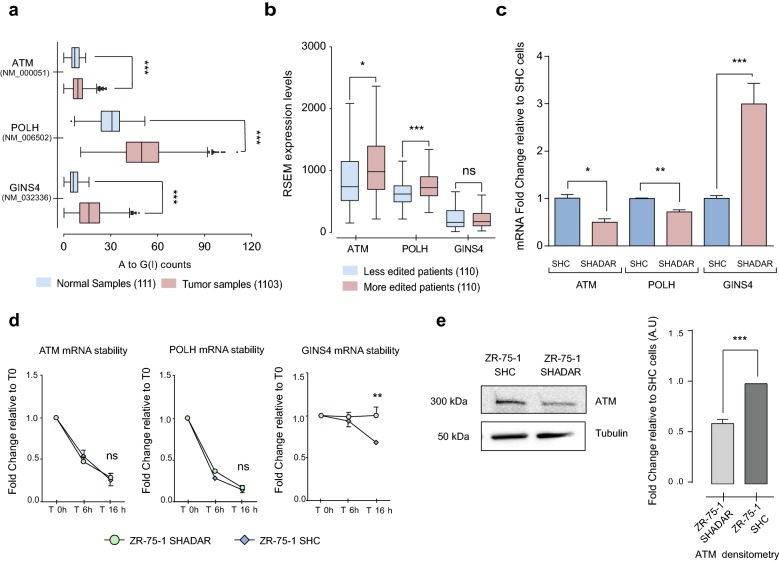
*ADAR1* knockdown induces expression changes on *ATM*, *GINS4* and *POLH* mRNAs. **a** Box and Whiskers plot for the A to G(I) counts present in normal (111) and tumoral samples (1103), in *ATM*, *GINS4* and *POLH* 3′ UTRs. **b** RSEM expression levels of p10 (lower deciles) and p90 (upper deciles) of edited tumors, based on A to G(I) counts, for *ATM*, *GINS4* and *POLH*. **c** mRNA expression levels of *ATM*, *POLH*, and *GINS4* in ZR-75-1 SHC and ZR-75-1 SHADAR cells. **d** *ATM*, *POLH*, and *GINS4* mRNA levels at 0, 6 and 16 h after Dactinomycin treatment (3 µg/mL). Results are expressed as relative to time 0. **e** Western Blot analysis for *ATM* expression levels in SHC and SHADAR cells. Quantification of 3 independent assays (right side). Two-way T-test: ***p < 0.001, **p < 0.0024, *p < 0.02, *ns* non-significant differences

Further, we compared the A to G(I) changes in the 571 UTRs in tumor and normal tissues. Remarkably, 114 UTR’s presented an increased editing number of editing site in the tumoral samples, compared to the normal samples analyzed (with at least 1.25 > FC), most of them consisting of 3′ UTRs (110/114), showing an increased level or number of edited sites of previously reported ADAR1 targets, including *APOL1* [23], *MDM2* [21], *MTDH* and *TNFAIP8L1* [24] (shown in Additional file 4). Interestingly, tumors show a significant increase number of edited sites at 3′UTRs of several important transcripts involved in gene expression related pathways such as metabolism of non-coding RNAs, generic transcription pathway, snRNP assembly and cell cycle, DNA damage response and DNA replication related pathways showing an increased number of edited sites on key mRNAs associated to that signaling pathways, such as *ATM*, *GINS4*, and *POLH* mRNAs (Fig. 3a). Remarkably, tumors from the highest editing counts decile, presents an higher *ATM*, and *POLH* expression compared to those tumors from the lower decile editing counts, (Fig. 3b), finding a significant correlation between *ADAR1* editing counts and the mRNA expression of *ATM* and *POLH* transcripts (Additional file 3: Table S2).

### *ADAR1* knockdown induces expression changes on *ATM*, *GINS4* and *POLH* mRNAs

Based on the correlations described above, we aimed to study the relation between *ADAR1* activity and the expression or stability of *ATM*, *GINS4* and *POLH*. To address this, a ZR-75-1 BC cell line was transduced with a lentivirus coding for a short hairpin RNA against *ADAR1* (ZR-75-1 SHADAR) or a scrambled sequence (ZR-75-1 SHC). ZR-75-1 cells were chosen because w*ild*-*type* (WT) ZR-75-1 cells show higher *ADAR1* expression and editing levels compared to MCF10A cells, measured using the RESS-qPCR assay (Additional file 5: Figure S2). ZR-75-1 SHADAR cells showed a significant down-regulation of *ATM* and *POLH* mRNA expression and an overexpression of *GINS4* mRNA levels, compared to ZR-75-1 SHC cells (Fig. 3c). Interestingly, there were no significant differences in the decay curves (after actynomicin D treatment) of *ATM* and *POLH* mRNA in ZR-75-1 SHADAR and SHC cells, whereas a significant increase in *GINS4* mRNA’s stability was found in ZR-75-1 SHADAR cells (Fig. 3d), indicating that editing of *GINS4* transcripts primarily modulates its stability, whereas editing of *ATM* and *POLH* primarily modulate their expression. *ATM* is a critical mediator of the DNA damage response and its expression was recently shown to be modulated by *ADAR1* in stressed cells [25]. Similarly, we confirmed that ZR-75-1 SHADAR cells present a significant decrease of total *ATM* protein expression compared to ZR-75-1 SHC cells (Fig. 3e).

**Fig. 4:**
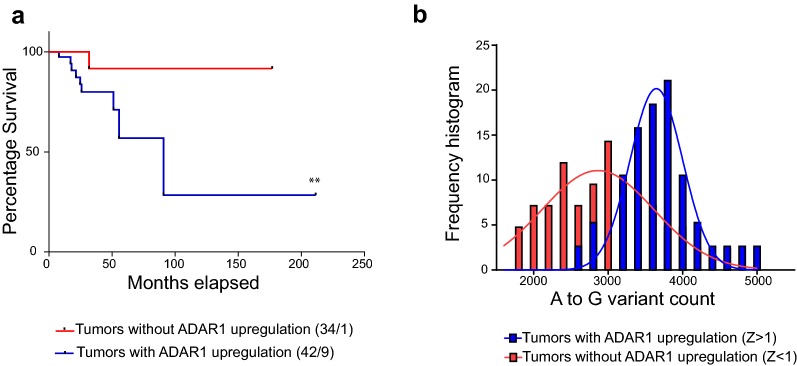
Clinical significance of A-to-I RNA editing in BRCA. **a** Kaplan-Maier survival proportions for basal-like patients stratified based on *ADAR1* mRNA expression levels. Basal-like patients with lower *ADAR1* expression are shown in red and Basal-like patients with highest *ADAR1* expression are shown in blue, respectively. **b** Histogram proportion for Basal-like TCGA tumors according to their A to G(I) variant count, showing the Basal-like patients selected for Kaplan-Maier analysis. Basal-like patients with lower *ADAR1* expression are shown in red and Basal-like patients with highest *ADAR1* expression are shown in blue, respectively. Gauss fit distribution for each subgroup is shown with a continuous line (red or blue accordingly). **a** Log-rank Mantel-Cox test ** < 0.0192 with a median survival of 90.75 months

Finally, we investigated if *ADAR* expression and activity could have a clinical association in BC patients. We found that high *ADAR1* expression is associated with shorter overall survival in patients with basal-like tumors (0.0192 Log-rank Mantel-Cox test; Fig. 4a); while no association was found in patients with tumors from the other PAM50 intrinsic BC subtypes (Additional file 3: Table S3). Interestingly, censored basal-like cancer patients with higher *ADAR1* expression pattern have a significant increase in the number of editing counts in their 571 selected UTRs, whereas patients with lower *ADAR1* expression showed lower edited sites (3683 ± 79.74 versus 2928 ± 103.3 edited sites, mean ± SEM, t test p < 0.0001, Fig. 4b). Considering these results is possible to suggest that basal-like cancer patients with more editing on their UTRs could have a lower overall survival.

## Discussion

In this work we analyzed the *ADAR* editing pattern across 81 breast cell lines, showing that *ADAR* A to G(I) edited sites correlates with both *ADAR* isoforms with catalytic activity, similar to other previous reports [17]. Noteworthy, in BC cells, there is an increased number of edited sites located at 3′UTR regions and exons, compared with non-tumoral cell lines, suggesting that 3′UTR sequence modification could be an important driving force for mRNA variability and regulation in the BC context. Our results from the BRCA TCGA UTRs analysis showed an overall increase in the number of edited sites in the tumoral samples compared to the normal samples of the cohort. Also, in the 1103 patients analyzed the editing counts of the 571 analyzed UTRs showed a strong correlation with *ADAR1* levels. Furthermore, our results show that Basal-like subtype BC patients with high level of *ADAR1* mRNA expression shows a worse clinical outcome and increased editing in their 3′UTRS, opening the possibility that the editing counts, present in the analyzed UTRs, could have a clinical significance. Given the intrinsic complexity of the edition process, further research is necessary to understand the association between OS and UTR edition. Nonetheless, these results complement the analysis of the relation between Alu edited sites and overall survival described in Paz-Yaacov et al. [26]. In addition, we found a significant correlation between the observed edited sites on 3′UTRs of *ATM* and *POLH* and their expression levels in the TCGA patients, suggesting that *ADAR1* could be involved in the mRNA expression or stability of these genes. To further test this relationship, we evaluated the expression and stability of these genes after *ADAR1* knockdown using ZR-75-1 cells. In agreement with the published work from Sakurai et al. [25], we found an overall diminution of both *ATM* mRNA and protein levels, after *ADAR1* knockdown.

The *ADAR1* mRNA editing implications for untranslated and intronic regions are an emerging area of investigation. It has been suggested that *ADAR1* function could have a tremendous impact on the mRNA target expression, stability and transcriptome variability [4]. In that line, 3′UTRs are an important regulatory structure of mRNA, that allow the interaction of different protein complexes with the mRNA, making them a platform to control mRNA stability, translation, and localization [27]. Moreover, 3′UTRs allow RNA/RNA interactions that function as an important regulatory mechanism for the regulation of mRNA expression. Recently, Qi et al. (2017) [24] reported that RNA editing in 3′UTRs undergo expression changes independently of their editing levels and *ADAR* dsRNA binding capabilities, suggesting that *ADAR1* could regulate the expression and/or stability of the editing target by a growing number of different mechanisms.

To date, few studies have focused on 3′UTR editing and its possible implication for cancer. A number of studies have looked for actionable coding mRNA edited sites that lead to phenotype changes such as viability, drug sensitivity, migration and proliferation in cellular cancer models [15, 17, 28–30]. However, increasingly more genome-wide analyses suggest that a large proportion of the A-to-I edited sites are located inside intronic and UTR regions, indicating that, in cancer, the deregulation of *ADAR1* involves a complex mechanism that may not be limited to A-to-I sites in this disease [4, 7, 20]. In this regard, recent reports suggest that the hyper-editing occurrence could be a marker for a more complex mechanism behind the *ADAR1* function, involving protein competition for binding sites or interacting partners, displacement of protein complexes and trans-regulatory mechanisms, such as increased processing/maturation of miRNAs. However, further studies are needed to understand the *ADAR1* function in UTRs in cancer, where a plethora of different mechanisms could explain the *ADAR1* role in target regulation in a cancer context.

### Conclusion

Taken together, our results show an increased number of edited sites and an increased level in the edited pool of shared sites present in 3′UTRs in BC cell lines and patients. This indicates that the editing function is mostly increased in this regulatory structure of the mRNA, affecting mRNAs associated with cancer.

## Methods

### RNA variant calling in BC cell lines dataset and data processing

Fastq files from 81 breast cell lines were obtained from Sequence Read Archive (SRA) PRJNA297219 BioProject [16] using SRA toolkit 2.5.7 software. Raw data is aligned to hg19 genome reference using STAR v2.4.2a software. BAM files are processed following the GATK’s best practices for RNA-seq. Variants were called using HaplotypeCaller (GATK v3.6) in default mode and annotated with dbSNP v147. Called edited sites were annotated by gene name, positional region and strand with UCSC genome annotation. RNA expression levels for all cell lines were calculated using HTseq (v0.6.1). For CNV and *ADAR1* correlation analysis, 49 breast cell lines CNV data was obtained from the CCLE database (https://portals.broadinstitute.org/ccle). Editing comparison for shared A to G(I) sites where made using a multi-sample VCF, comparing each BC cell line against each immortalized non-malignant cell line (MCF10A, MCF12 and 184A1), to further perform a Fisher exact test to analyze the differences at site level and a Wilcoxon test across all shared sites on a particular transcript region, to test the enrichment of a particular region on BC cells.

### RNA variant calling in UTRs using the TCGA dataset and data processing

The UTRs of interest were chosen as follows: First, RNA edited sites described in previously reported studies [5, 6, 19] were selected to further intersect the variant list and only those UTR regions (based on Refseq coordinates) with variants were kept according to this intersected variant list. Then, the filtered UTRs list was intersected with the RADAR [31] database, to finally include only those UTRs with at least one previously validated editing site to keep them for further analysis. Reads overlapping the UTRs of interest were downloaded from the Cancer Genomics Hub (CGHub) using bam-slicer. Then, the reads were processed following the GATK’s best practices for RNA-seq, and variants were called using UnifiedGenotyper in default mode. Finally, RNA variants present in dbSNP v138 were annotated as SNPs, and the remaining ones as candidate edited sites. In addition, previously validated data from Wang, et al. [5], and RepeatMasker database were used in order to analyze and characterize the called variants. For further analysis, such as variation count, vcftools (v0.1.13), vcf-query and Unix command line tools were used. All the data generated was directly processed in R (3.2.0) or Unix command line.

### Gene ontology and pathway enrichment analysis

Gene ontology enrichment was carried out using Cytoscape (v3.0.1) software and the ClueGO (v3.3.3) plugin. Briefly, a gene list from each analysis was submitted on this software using Reactome pathway enrichment db for further comparisons. For breast cell lines GO enrichment analysis we used the gene list derivate from the edited sites present at least in 10% of 81 breast cells. Only statistically significant groups were displayed, using a Bonferroni step-down multiple comparison post hoc test. Corrected p < 0.05 was considered statistically significant. For the cell lines analysis, the set of breast cell lines expressed genes were used as a background, using genes with more than Log_2_(1) FPKM values present in more than 10% (8) of the samples.

### Survival analysis

Overall survival data from BRCA TCGA patients belonging to specific PAM50 molecular subgroups was obtained from cBioPortal [32] using the clinical data from TCGA. Tumors were stratified according to their *ADAR* mRNA expression levels into |z| > 1 and |z| < 1 groups. Kaplan Maier survival curves were plotted and analyzed in GraphPad Prism 7.0. A log-rank test, p < 0.05 was considered significant.

### Cell culture

MCF10A, MCF7 and ZR-75-1 breast cell lines were obtained from ATCC and cultured under standard conditions, at 37 °C in a humidified incubator containing 5% CO_2_. Cells were routinely tested for mycoplasma contamination using the PCR Mycoplasma Test Kit EZ-PCR (Biological Industries) following the manufacturer’s instructions.

### Lentiviral transduction

ZR-75-1 cells were transduced with a commercial pre-package lentiviral vector (GeneTarget, LTSH-U6-RP) coding a shRNA against *ADAR1* mRNA or a scrambled ShRNA (GeneTarget U6(shRNA-Ctr)-RP) as a control. The *ADAR1* shRNA sequence corresponds to the TRCN0000050789 sequence obtained from The RNAi consortium. Cells were maintained in growth media with 1 µg/mL Puromycin (Invitrogen) for selection to further sort them.

### Immunoblotting

Protein lysates and western blot were processed as described in Sagredo et al. (2017) [33]. Briefly, protein lysates were generated using RIPA buffer (25 mM Tris–HCl pH 7.6, 150 mM NaCl, 5 mM EDTA, 1% v/v TritonX-100, 1% w/v sodium deoxycholate, 0.1% w/v SDS) and protease (Calbiochem) and phosphatase (Roche Life Science) inhibitor cocktails. Protein lysates (30 µg per lane) were resolved in 8% sodium dodecyl sulfate–polyacrylamide gel electrophoresis (SDS-PAGE) and proteins were transferred onto a nitrocellulose membrane. Membranes were blocked in 5% w/v BSA (Winkler), and then incubated with primary antibodies at 4 °C overnight. Anti-ADAR1 (Cell signaling, 14,175) Rabbit anti-ATM (Cell Signaling, 2873) and mouse anti-α-tubulin (Sigma-Aldrich, T5168) antibodies were used. All primary antibodies were detected using appropriate HRP-conjugated secondary antibodies and a chemiluminescence reagent (SuperSignal West Pico Chemiluminescent Substrate, Thermo Scientific). Finally, images were obtained using the ChemiScope3500 Mini chemiluminescence imaging system (Clinx Science Instruments).

### RNA extraction, quantitative PCR and RNA stability assay

RNA extraction, cDNA synthesis and quantitative PCR were processed as described in Sagredo et al. [33]. ACTB was used as a housekeeping gene. In addition, RESS-qPCR assays for AZIN1 and MDM2 targets were performed according to Crews et al. [34]. The complete primer list is presented in Additional file 6. mRNA stability of ATM, GINS4 and POLH, in ZR-75-1 SHC and SHADAR cells were assessed using Dactinomycin (SIGMA-Aldrich) (3 µg/mL) at 0, 6, 8 and 16 h, and total RNA was extracted to analyze the different gene of interest using qRT-PCR. To compare the mRNA decay curves, each condition was normalized against the respective group of 0 h treated cells.

### PCR and Sanger sequencing

For *ATM*, *AZIN1* and *MDM2* 3′UTR sequences, PCR reactions were performed using the High fidelity Phusion polymerase (Thermo Fisher Scientific) following the manufacturer’s instructions. PCR products were run in an electrophoretic gel and purified with the Wizard PCR clean-up kit (Promega). Chromatogram results were analyzed using CLC Main Workbench v5.5 (CLC Bio). *ATM* 3′UTR primers were previously described by Wang et al. [5].

### Statistical analysis

Statistical analysis was performed using GraphPad Prism 7.0. qRT-PCR and Western Blot were examined by Two tailed Student’s t-test. For each comparison p < 0.05 was considered statistically significant, with at least 3 independent experiments performed for each analysis.

## Additional files

**Additional file 1.** A to G(I) variants present on breast cell lines.

**Additional file 2.** A to G (I) counts comparison between normal Cells and BC cells. **Figure S1A.** A to G (I) counts comparison between normal cells (3) and BC cells (78). **Figure S1B.** Editing level comparison between normal and BC cells for those shared variants located at 3′UTRs.

**Additional file 3.** Supplementary tables associated to SRA and TCGA BRCA analysis.

**Additional file 4.** UTRs with an increased editing number on BC samples.

**Additional file 5.** ZR-75-1 cell line characterization.

**Additional file 6.** List of primer sequences used for qRT-PCR and RESS-qPCR.

## Abbreviations

ADAR: adenosine deaminases acting on RNA
BRCA TCGA: Breast Invasive Carcinoma Cancer Genome Atlas
UTRs: untranslated regions
VCF: variant calling file
RNA: ribonucleic acid
shRNA: short harping RNA
mRNA: messenger RNA
A-to-I: adenosine to inosine
CNV: copy number variation
BC: breast cancer
miRNAs: microRNAs

## References

[1] Keegan LP, Gallo A, O’Connell MA (2001). The many roles of an RNA editor. Nat Rev Genet.

[2] Chen L, Li Y, Lin CH, Chan THM, Chow RKK, Song Y, Liu M, Yuan YF, Fu L, Kong KL, Qi L, Li Y, Zhang N, Tong AHY, Kwong DLW, Man K, Lo CM, Lok S, Tenen DG, Guan XY (2013). Recoding RNA editing of AZIN1 predisposes to hepatocellular carcinoma. Nat Med.

[3] Chan THM, Lin CH, Qi L, Fei J, Li Y, Yong KJ, Liu M, Song Y, Chow RKK, Ng VHE, Yuan YF, Tenen DG, Guan XY, Chen L (2014). A disrupted RNA editing balance mediated by ADARs (Adenosine DeAminases that act on RNA) in human hepatocellular carcinoma. Gut.

[4] Bahn JH, Ahn J, Lin X, Zhang Q, Lee JH, Civelek M, Xiao X (2015). Genomic analysis of ADAR1 binding and its involvement in multiple RNA processing pathways. Nat. Commun..

[5] Wang IX, So E, Devlin JL, Zhao Y, Wu M, Cheung VG (2013). ADAR regulates RNA editing transcript stability, and gene expression. Cell Rep..

[6] Peng Z, Cheng Y, Tan BC-M, Kang L, Tian Z, Zhu Y, Zhang W, Liang Y, Hu X, Tan X, Guo J, Dong Z, Liang Y, Bao L, Wang J (2012). Comprehensive analysis of RNA-Seq data reveals extensive RNA editing in a human transcriptome. Nat Biotechnol.

[7] Galipon J, Ishii R, Suzuki Y, Tomita M, Ui-Tei K (2017). Differential binding of three major human ADAR isoforms to coding and long non-coding transcripts. Genes..

[8] Maas S, Godfried Sie CP, Stoev I, Dupuis DE, Latona J, Porman AM, Evans B, Rekawek P, Kluempers V, Mutter M, Gommans WM, Lopresti D (2011). Genome-wide evaluation and discovery of vertebrate A-to-I RNA editing sites. Biochem Biophys Res Commun.

[9] Tan MH, Li Q, Shanmugam R, Piskol R, Kohler J, Young AN, Liu KI, Zhang R, Ramaswami G, Ariyoshi K, Gupte A, Keegan LP, George CX, Ramu A, Huang N, Pollina EA, Leeman DS, Rustighi A, Goh YPS, Chawla A, Del Sal G, Peltz G, Brunet A, Conrad DF, Samuel CE, O’Connell MA, Walkley CR, Nishikura K, Li JB (2017). Dynamic landscape and regulation of RNA editing in mammals. Nature.

[10] Rueter SM, Dawson TR, Emeson RB (1999). Regulation of alternative splicing by RNA editing. Nature.

[11] Goldberg L, Abutbul-Amitai M, Paret G, Nevo-Caspi Y (2017). Alternative splicing of STAT3 is affected by RNA editing. DNA Cell Biol..

[12] Zhang Z, Carmichael GG (2001). The fate of dsRNA in the nucleus: a p54nrb-containing complex mediates the nuclear retention of promiscuously A-to-I edited RNAs. Cell.

[13] Liscovitch N, Bazak L, Levanon EY, Chechik G (2014). Positive correlation between ADAR expression and its targets suggests a complex regulation mediated by RNA editing in the human brain. RNA Biol.

[14] Stellos K, Gatsiou A, Stamatelopoulos K, Perisic Matic L, John D, Lunella FF, Jaé N, Rossbach O, Amrhein C, Sigala F, Boon RA, Fürtig B, Manavski Y, You X, Uchida S, Keller T, Boeckel JN, Franco-Cereceda A, Maegdefessel L, Chen W, Schwalbe H, Bindereif A, Eriksson P, Hedin U, Zeiher AM, Dimmeler S (2016). Adenosine-to-inosine RNA editing controls cathepsin S expression in atherosclerosis by enabling HuR-mediated post-transcriptional regulation. Nat Med.

[15] Amin EM, Liu Y, Deng S, Tan KS, Chudgar N, Mayo MW, Sanchez-Vega F, Adusumilli PS, Schultz N, Jones DR (2017). The RNA-editing enzyme ADAR promotes lung adenocarcinoma migration and invasion by stabilizing FAK. Sci Signal..

[16] Marcotte R, Sayad A, Brown KR, Sanchez-Garcia F, Reimand J, Haider M, Virtanen C, Bradner JE, Bader GD, Mills GB, Pe’er D, Moffat J, Neel BG (2016). Functional genomic landscape of human breast cancer drivers, vulnerabilities, and resistance. Cell.

[17] Fumagalli D, Gacquer D, Rothé F, Lefort A, Libert F, Brown D, Kheddoumi N, Shlien A, Konopka T, Salgado R, Larsimont D, Polyak K, Willard-Gallo K, Desmedt C, Piccart M, Abramowicz M, Campbell PJ, Sotiriou C, Detours V (2015). Principles governing A-to-I RNA editing in the breast cancer transcriptome. Cell Rep..

[18] Lazzari E, Mondala PK, Santos ND, Miller AC, Pineda G, Jiang Q, Leu H, Ali SA, Ganesan A-P, Wu CN, Costello C, Minden M, Chiaramonte R, Stewart AK, Crews LA, Jamieson CHM (2017). Alu-dependent RNA editing of GLI1 promotes malignant regeneration in multiple myeloma. Nat. Commun..

[19] Ramaswami G, Lin W, Piskol R, Tan MH, Davis C, Li JB (2012). Accurate identification of human Alu and non-Alu RNA editing sites. Nat Methods.

[20] Chen T, Xiang JF, Zhu S, Chen S, Yin QF, Zhang XO, Zhang J, Feng H, Dong R, Li XJ, Yang L, Chen LL (2015). ADAR1 is required for differentiation and neural induction by regulating microRNA processing in a catalytically independent manner. Cell Res.

[21] Zhang L, Yang CS, Varelas X, Monti S (2016). Altered RNA editing in 3′ UTR perturbs microRNA-mediated regulation of oncogenes and tumor-suppressors. Sci Rep..

[22] Yang C-C, Chen Y-T, Chang Y-F, Liu H, Kuo Y-P, Shih C-T, Liao W-C, Chen H-W, Tsai W-S, Tan BC-M (2017). ADAR1-mediated 3′ UTR editing and expression control of antiapoptosis genes fine-tunes cellular apoptosis response. Cell Death Dis..

[23] Sharpnack MF, Chen B, Aran D, Kosti I, Sharpnack DD, Carbone DP, Mallick P, Huang K (2017). Global transcriptome analysis of RNA abundance regulation by ADAR in lung adenocarcinoma. EBioMedicine..

[24] Qi L, Song Y, Chan THM, Yang H, Lin CH, Tay DJT, Hong HQ, Tang SJ, Tan KT, Huang XX, Lin JS, Ng VHE, Maury JJP, Tenen DG, Chen L (2017). An RNA editing/dsRNA binding-independent gene regulatory mechanism of ADARs and its clinical implication in cancer. Nucleic Acids Res.

[25] Sakurai M, Shiromoto Y, Ota H, Song C, Kossenkov AV, Wickramasinghe J, Showe LC, Skordalakes E, Tang HY, Speicher DW, Nishikura K (2017). ADAR1 controls apoptosis of stressed cells by inhibiting Staufen1-mediated mRNA decay. Nat Struct Mol Biol.

[26] Paz-Yaacov N, Bazak L, Buchumenski I, Porath HT, Danan-Gotthold M, Knisbacher BA, Eisenberg E, Levanon EY (2015). Elevated RNA editing activity is a major contributor to transcriptomic diversity in tumors. Cell Rep..

[27] Castello A, Fischer B, Hentze MW, Preiss T (2013). RNA-binding proteins in Mendelian disease. Trends Genet.

[28] Han L, Diao L, Yu S, Xu X, Li J, Zhang R, Yang Y, Werner HMJ, Eterovic AK, Yuan Y, Li J, Nair N, Minelli R, Tsang YH, Cheung LWT, Jeong KJ, Roszik J, Ju Z, Woodman SE, Lu Y, Scott KL, Li JB, Mills GB, Liang H (2015). The genomic landscape and clinical relevance of A-to-I RNA editing in human cancers. Cancer Cell.

[29] Song C, Sakurai M, Shiromoto Y, Nishikura K (2016). Functions of the RNA editing enzyme ADAR1 and their relevance to human diseases. Genes..

[30] Gumireddy K, Li A, Kossenkov AV, Sakurai M, Yan J, Li Y, Xu H, Wang J, Zhang PJ, Zhang L, Showe LC, Nishikura K, Huang Q (2016). The mRNA-edited form of GABRA3 suppresses GABRA3-mediated Akt activation and breast cancer metastasis. Nat. Commun..

[31] Ramaswami G, Li JB (2014). RADAR: a rigorously annotated database of A-to-I RNA editing. Nucleic Acids Res.

[32] Gao J, Aksoy BA, Dogrusoz U, Dresdner G, Gross B, Sumer SO, Sun Y, Jacobsen A, Sinha R, Larsson E, Cerami E, Sander C, Schultz N (2013). Integrative analysis of complex cancer genomics and clinical profiles using the cBioPortal. Sci. Signal..

[33] Sagredo AI, Sagredo EA, Cappelli C, Báez P, Rodrigo AM, Blanco C, Tapia JC, Echeverría C, Cerda O, Stutzin A, Simon F, Marcelain K, Armisén R (2017). TRPM4 regulates Akt/GSK3-β activity and enhances β-catenin signaling and cell proliferation in prostate cancer cells. Mol Oncol..

[34] Crews LA, Jiang Q, Zipeto MA, Lazzari E, Court AC, Ali S, Barrett CL, Frazer KA, Jamieson CHM (2015). An RNA editing fingerprint of cancer stem cell reprogramming. J Transl Med..

