# Supplementary material for: ADAR1-mediated RNA-editing of 3′UTRs in breast cancer
Source: Biol Res. 2018 Oct 5;51:36. doi: 10.1186/s40659-018-0185-4 (PMC6172785; doi:10.1186/s40659-018-0185-4)
Supplement: Supplementary file 2 — Additional file 2. A to G (I) counts comparison between normal Cells and BC cells. Figure S1A. A to G (I) counts comparison between normal cells (3) and BC cells (78). Figure S1B. Editing level comparison between normal and BC cells for those shared variants located at 3′UTRs. [file 40659_2018_185_MOESM2_ESM.pdf]

Additional File 2.

A

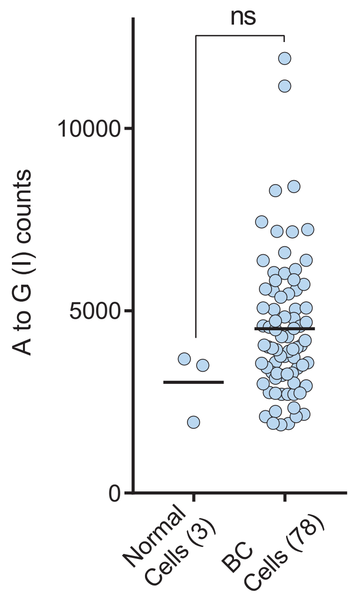

Figure S1A. A to G (I) counts comparison between Normal Cells (3) and BC cells (78). ns: non-significant differences, Kolmogorov-Smirnov Test.

B

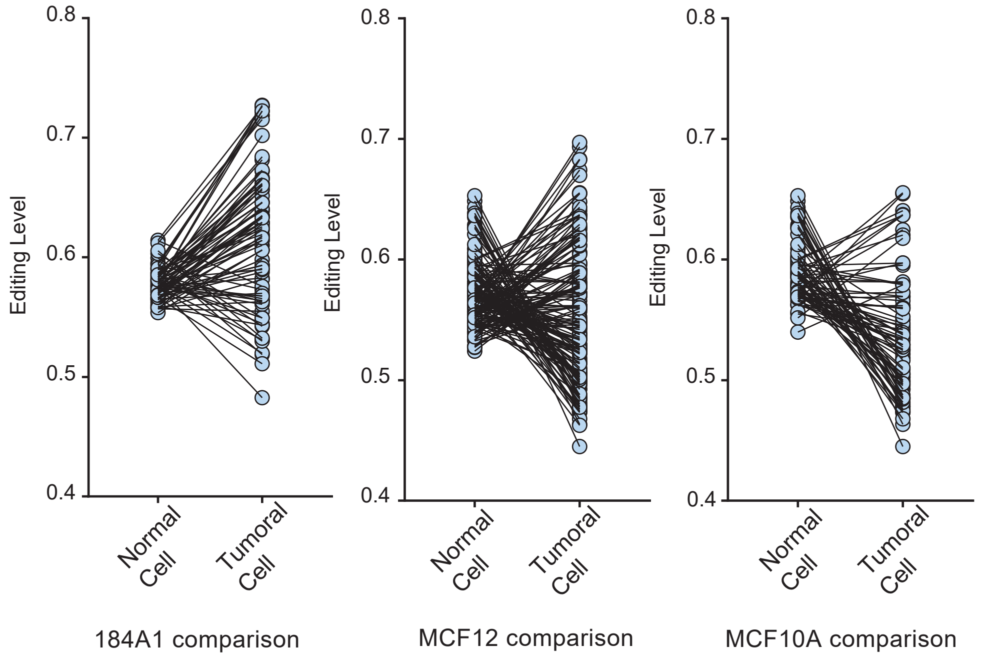

Figure S1B. Editing level comparison between normal and BC cells for those shared variants located at 3'UTRs. Displayed the mean editing level for each paired comparisons
