# Supplementary material for: ADAR1-mediated RNA-editing of 3′UTRs in breast cancer
Source: Biol Res. 2018 Oct 5;51:36. doi: 10.1186/s40659-018-0185-4 (PMC6172785; doi:10.1186/s40659-018-0185-4)
Supplement: Supplementary file 3 — Additional file 3. Supplementary tables associated to SRA and TCGA BRCA analysis. [file 40659_2018_185_MOESM3_ESM.pdf]

Table S1.

	3UTR	5UTR	Exons	introns
**184A1**	52	5	1	7
**MCF10A**	9	4	1	4
**MCF12**	44	19	2	37

Table S2.

Target ID	r	P (two-tailed)
**POLH**	0.301	< 0.0001
**ATM**	0.244	0.0002
**GINS4**	0.0516	0.4442

Table S3.

**LumA**	**TOTAL CASES**	**DECEASED**	**MEDIAN MONTHS SURVIVAL**
Cases with Alteration(s) in Query Gene(s)	97	9	129.61
Cases without Alteration(s) in Query Gene(s)	138	17	113.74
			Logrank Test P: 0.630
**LumB**	**TOTAL CASES**	**DECEASED**	**MEDIAN MONTHS SURVIVAL**
Cases with Alteration(s) in Query Gene(s)	67	10	81.58
Cases without Alteration(s) in Query Gene(s)	64	7	79.57
			Logrank Test P: 0.894

Table S1. Significative comparisons (Wilcoxon Test p<0.05) between BC cells and interrogated immortalized cell line for those shared variants located in different transcript regions.

Table S2. Pearson Correlation for *ATM*, *GINS4* and *POLH* expression and editing counts.

Table S3. Survival proportions for LumA and LumB BRCA TCGA patients based on ADAR1 expression. Data obtained from The Cancer Genome Atlas Network. Comprehensive molecular portraits of human breast tumors. Nature 490, 61–70 (2012).
