# Supplementary material for: ADAR1-mediated RNA-editing of 3′UTRs in breast cancer
Source: Biol Res. 2018 Oct 5;51:36. doi: 10.1186/s40659-018-0185-4 (PMC6172785; doi:10.1186/s40659-018-0185-4)
Supplement: Supplementary file 5 — Additional file 5. ZR-75-1 cell line characterization. [file 40659_2018_185_MOESM5_ESM.pdf]

Additional File 5.

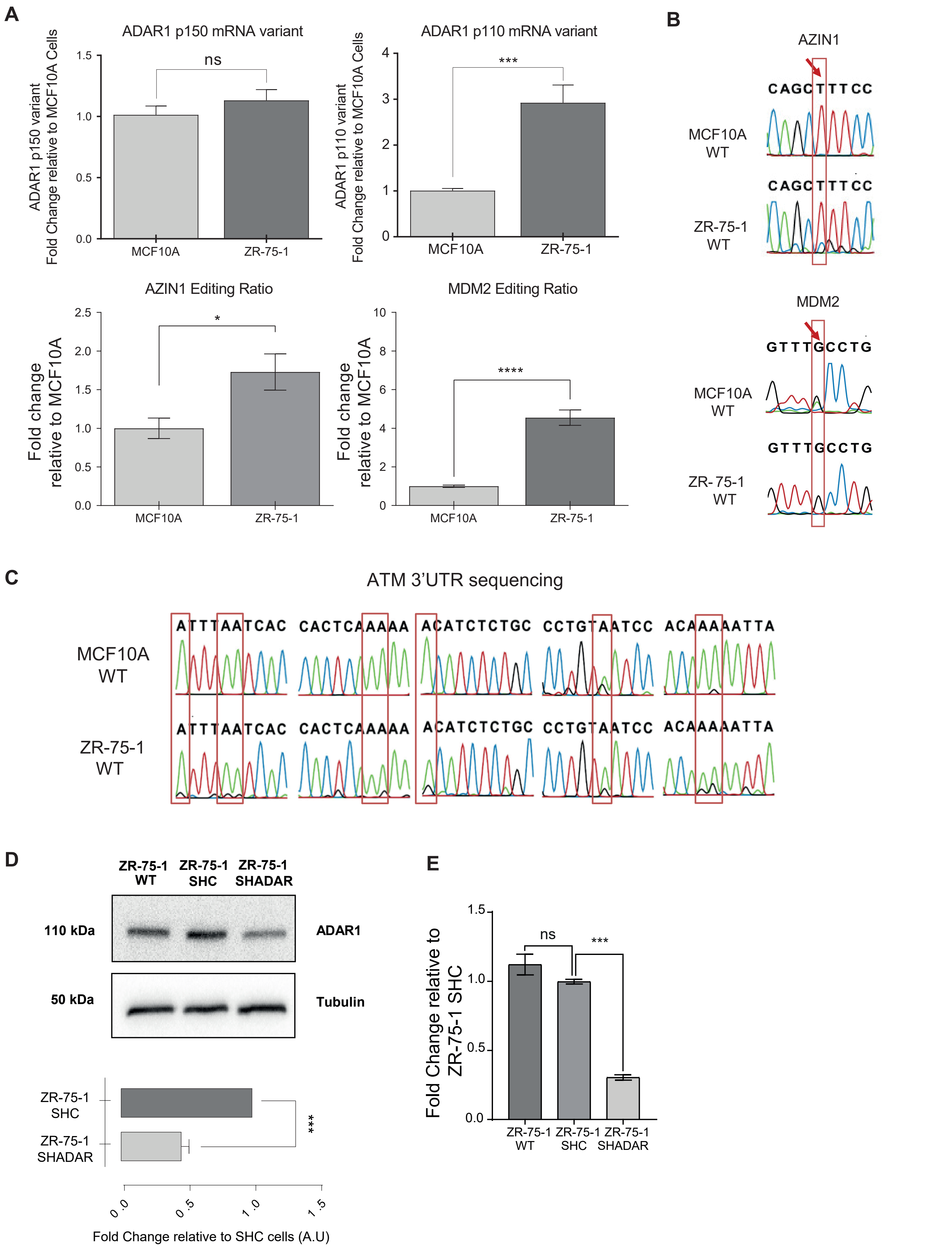

Figure S2. Breast cancer cell lines characterization. (A) ADAR1 mRNA variants expression for MCF10A and ZR-75-1 wild type cells (upper panel) and RESS-qPCR for MCF10A and ZR-75-1 wild type cells for AZIN1 and MDM2 positions (lower panel). (B) MDM2 and AZIN1 electropherogram analysis for PCR products evaluated in (A). (C) Electropherogram analysis for ATM 3'UTR evaluated in MCF10A and ZR-75-1 wild type cells, based on Wang, et al. (2013) [5]. (D) ADAR1 protein expression in ZR-75-1 WT, SHC and SHADAR cells. Down, quantification of 3 independent assays. (E) qRT-PCR analysis for ADAR1 expression in ZR-75-1 WT, ZR-75-1 SHC and ZR-75-1 SHADAR cells. At least 3 independent experiments were performed in each experiment. Two-way T-test. ****<0.0001,***<0.001, **<0.01, *<0.05, ns: non-significant differences.
